# Expression/activation of α5β1 integrin is linked to the β-catenin signaling pathway to drive migration in glioma cells

**DOI:** 10.18632/oncotarget.11552

**Published:** 2016-08-23

**Authors:** Guillaume Renner, Fanny Noulet, Marie-Cécile Mercier, Laurence Choulier, Nelly Etienne-Selloum, Jean-Pierre Gies, Maxime Lehmann, Isabelle Lelong-Rebel, Sophie Martin, Monique Dontenwill

**Affiliations:** ^1^ UMR7213 CNRS, Team Tumoral Signaling and Therapeutic Targets, Université de Strasbourg, Faculté de Pharmacie, Illkirch, France

**Keywords:** α5β1 integrin, beta-catenin, migration, EMT, glioma

## Abstract

The Wnt/beta catenin pathway has been highlighted as an important player of brain tumors aggressiveness and resistance to therapies. Increasing knowledges of the regulation of beta-catenin transactivation point out its hub position in different pathophysiological outcomes in glioma such as survival and migration. Crosstalks between integrins and beta-catenin pathways have been suggested in several tumor tissues. As we demonstrated earlier that α5β1 integrin may be considered as a therapeutic target in high grade glioma through its contribution to glioma cell migration and resistance to chemotherapy, we addressed here the potential relationship between α5β1 integrin and beta-catenin activation in glioma cells. We demonstrated that overexpression and activation by fibronectin of α5β1 integrin allowed the transactivation of beta-catenin gene targets included in an EMT-like program that induced an increase in cell migration. Hampering of beta catenin activation and cell migration could be similarly achieved by a specific integrin antagonist. In addition we showed that α5β1 integrin/AKT axis is mainly involved in these processes. However, blockade of beta-catenin by XAV939 (tankyrase inhibitor leading to beta-catenin degradation) did not synergize with p53 activation aiming to cell apoptosis as was the case with integrin antagonists. We therefore propose a dual implication of α5β1 integrin/AKT axis in glioma cell resistance to therapies and migration each supported by different signaling pathways. Our data thus suggest that α5β1 integrin may be added to the growing list of beta-catenin modulators and provide new evidences to assign this integrin as a valuable target to fight high grade glioma.

## INTRODUCTION

Since several years association of the canonical Wnt/beta-catenin signaling pathways with tumorigenesis and tumor aggressiveness has garnered significant attention in different solid tumors thus leading to the development of potential inhibitors [1–3]. Its aberrant activation has been reported in glioma, and Wnt pathway component expressions have been correlated with histological grades of malignancy [4–7]. Dysregulation of Wnt/beta-catenin pathway was shown to contribute to glioma development and progression [8] as well as invasion [9]. Studies have indicated that binding of wnts (wnt1, wnt3a; [10]) to receptors of the Frizzled/LRP families, leads to beta-catenin evasion from GSK3β-induced degradation, accumulation in the cytoplasm, translocation in the nucleus and transcription of various Wnt target genes involved in proliferation (c-myc, cyclin D1, ..) or negative regulators of the pathway (axin, DKK1..). Beside its participation in canonical Wnt pathway, beta-catenin is also an integral part of the cadherin-catenin complexes at the cell membranes which drive the cell-cell contacts. Likewise, the disruption of the interactions between cadherin, alpha-catenin and beta-catenin has been shown to lead to translocation in the nucleus and transactivation of beta-catenin together with increased migration [8]. Activation of cell membrane-associated kinases (growth factor receptors such as EGFR, which is known to affect pathogenesis and prognosis of glioma) is thought to disrupt these complexes and to promote beta-catenin transactivation and cell migration [11] independently of the Wnt pathway [12] although crosstalks may exist between them [13]. Beta-catenin thus appears as an important hub/determinant in glioma aggressiveness controlling both proliferation/survival and migration/invasion of tumoral cells. Very recently, upregulated Wnt/beta-catenin signaling was reported to induce radioresistance [14] and chemoresistance [15] in glioma.

Due to the complexity of the networks involving beta-catenin and its major contribution to the pathology, the search for unknown drivers remains an important topic in the field of glioma. We and others have previously characterized the α5β1 integrin, also known as the fibronectin receptor, as playing a key role in the pathobiology of glioma [16, 17]. Integrins are membrane-localised protein heterodimers recognizing specific extracellular matrix components and integrating the microenvironmental cues into signaling pathways. They are involved in many tumoral processes [18] and particularly in survival, migration and resistance to therapies. We previously demonstrated that specific α5β1 integrin antagonists block fibronectin-induced cell migration [19] and sensitize glioma cells to chemotherapy in a p53 status-dependent way [16, 20, 21]. Crosstalks between integrin and Wnt/beta-catenin pathways have been suggested first in non tumoral cells and more recently in solid tumors. In general, activation of integrins induces downstream protein kinases activation (ILK, FAK..) paralleling with destabilization of cadherin/beta-catenin complexes dependently or independently from the Wnt pathway [22–24]. Transcriptional activity of beta-catenin in the nucleus results in an epithelial to mesenchymal (EMT) program and increases cell migration [25].

To date, very few data concerning an interconnection between α5β1 integrin and beta-catenin activity are available [26] and, to our knowledge, none for tumors (including glioma). The aim of this study was to explore the potential link between the expression/activation of α5β1 integrin and beta-catenin transcriptional activity in glioma cell lines and to investigate the phenotypical consequences.

## RESULTS

### Integrin α5β1 expression and activation induce β-catenin activation

**Figure 1:**
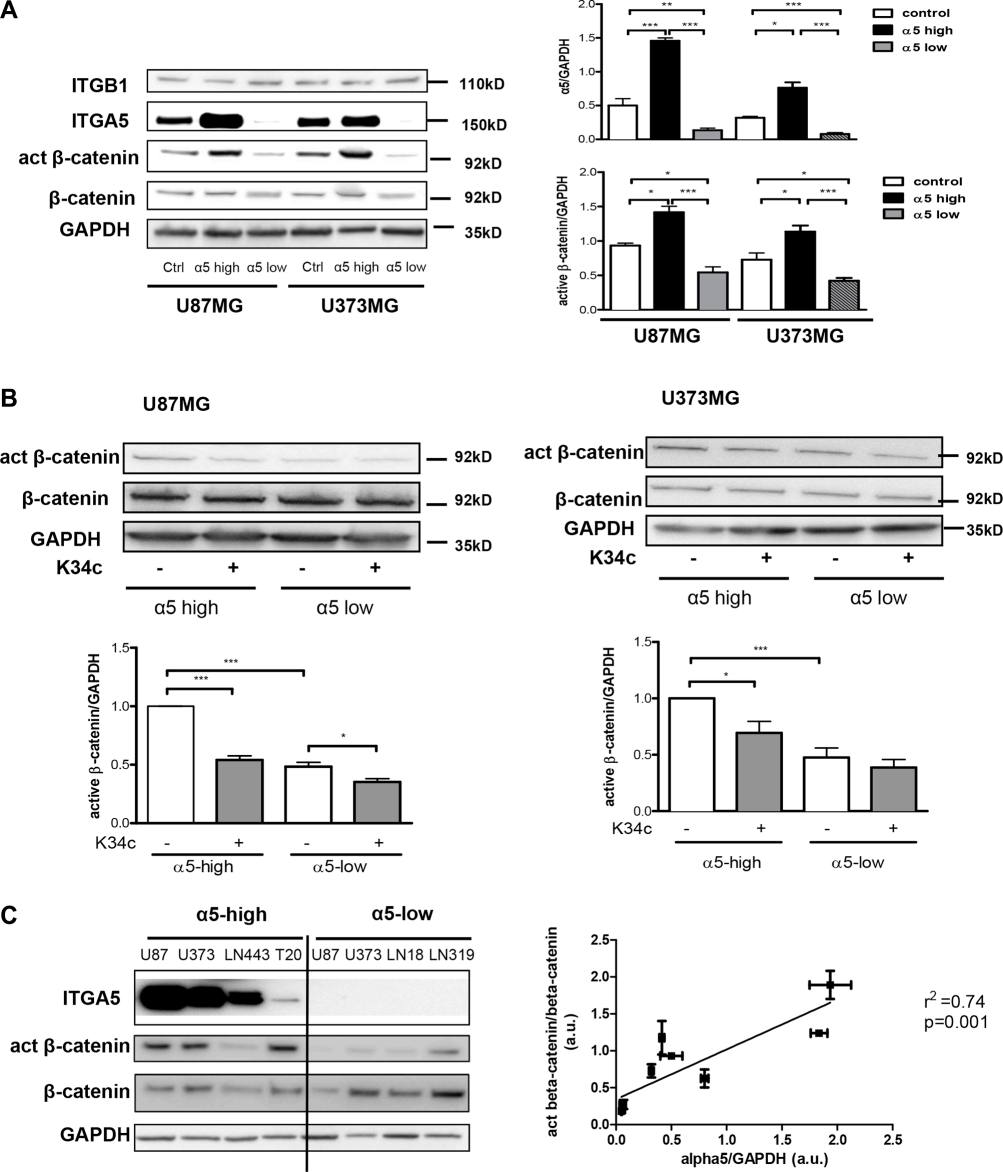
Expression of α5β1 integrin is related to beta-catenin activation (**A**) Western blot analysis of α5 integrin subunit (ITGA5), β1 integrin subunit (ITGB1), active beta-catenin (act β-catenin) and total beta-catenin (β-catenin) expressions in U87MG and U373MG control cells (transfected with the empty vector), overexpressing (α5-high) or repressed for (α5-low) α5 integrin. (**B**) Western blot analysis of active beta-catenin and total beta-catenin level after 90-min treatment with K34c integrin antagonist (20 μM). (**C**) Left. Relationships between α5β1 integrin levels and β-catenin activation in different glioma cell lines. One western blot representative of 3 independent experiments is shown. Right. Linear regression analysis of the relationship occurring between α5 expression level and active beta-catenin using data from the western blot in b) and values obtained from control cells in western blot of a). Histograms represent the mean ± S.E.M. of 3 independent experiments normalized with GAPDH with *p < 0,05; **p < 0,01; ***p < 0,005.

We first determined whether β-catenin activation could be linked to α5β1 expression in U87MG and U373MG glioma cell lines. Measure of the beta-catenin basal level of activation was achieved with cells deprived of serum for 90 min as described in Material and Methods. Activation of beta-catenin was recorded with a specific antibody which recognizes a non-phosphorylated Ser37/Thr41 N-terminal beta-catenin epitope known to be generated upon Wnt signaling or GSK3β pharmacological inhibition [27]. As shown in the Figure 1A, in the absence of exogenous stimuli, expression of α5 integrin is clearly related to the expression of this activated form of beta-catenin in both cell lines. We also investigated the effect of a specific α5β1 integrin inhibitor K34c that proved able to inhibit U87MG cell survival and migration [19]. Under these basal conditions, K34c inhibited the activated form of beta-catenin in α5-high cells to the level of α5-low cells with little impact on α5-low cells (Figure 1B). Data thus suggested that in basal conditions, expression of α5 integrin was linked to beta-catenin activation in U87MG and U373MG cells which could be inhibited by a specific α5β1 integrin antagonist. The relationship between α5 integrin level and beta-catenin activation was evaluated in other glioma cell lines as shown in the Figure 1C. Data from western blot analysis (Figure 1C, left) indicated a significant correlation between these two parameters when 6 glioma cell lines were considered (Figure 1C, right).

**Figure 2:**
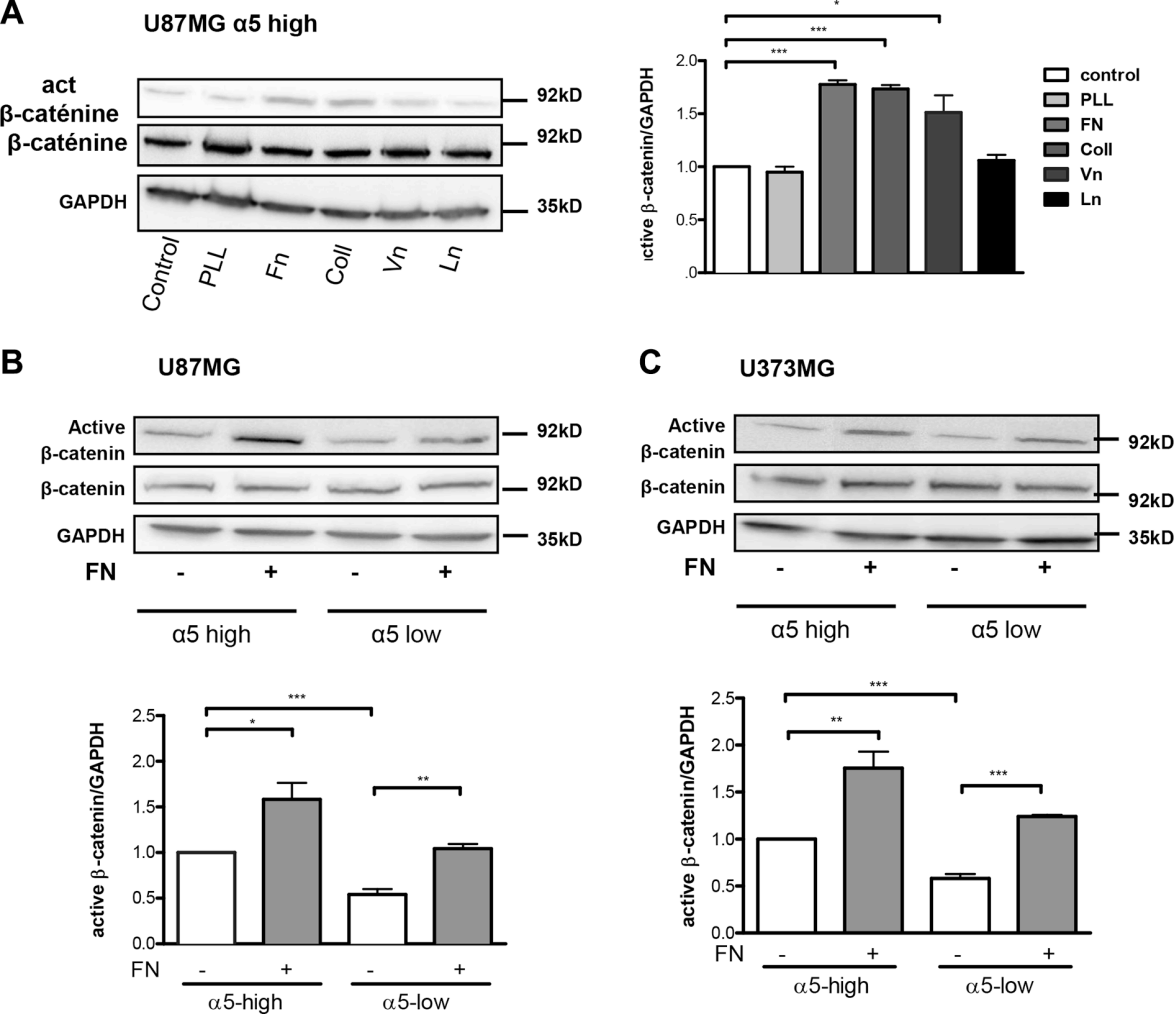
Fibronectin matrix triggers active β-catenin (**A**) Western blot analysis of β-catenin activation in U87MG-α5 high cells plated for 90-min on uncoated (control) or 10 μg/ml poly-L-lysine (PLL), fibronectin (Fn), collagen (Coll), vitronectin (Vn) or laminin (Ln) coated wells. GAPDH was used as a loading control. (**B**) Western blot analysis of fibronectin-induced effects on activation of β-catenin in U87MG α5-high and α5-low cells. Cells were plated on fibronectin (10 μg/ml)-coated wells for 90-min. (**C**) Similar experiments as in b) for U373MG α5-high and α5-low cells. One western blot representative of 3 independent experiments is shown. Histograms represent the mean ± S.E.M. of 3 independent experiments normalized with GAPDH with *p < 0,05; **p < 0,01; ***p < 0,005.

We then analyzed if α5β1 integrin activation through binding to fibronectin may enhance beta-catenin activation. For this purpose, U87MG-α5 high cells were plated on fibronectin pre-coated wells. The effects of other ECM components (collagen, vitronectin, laminin) were compared to those obtained on non-coated or with poly-L-lysine (a non ECM component) coated wells. As compared to uncoated wells, poly-L-lysine and laminin did not improve the active beta-catenin fraction in U87MG-α5 high cells (Figure 2A) thus ruling out a role of laminin receptors (α1β4, α6β4). However, likewise to the increase induced by fibronectin, collagen and vitronectin were both able to similarly enhance the beta-catenin activity suggesting a role of collagen-binding β1 integrins and vitronectin-binding αv integrins on these substrates. Our data are in agreement with other studies on non-glioma cells showing that collagen- or vitronectin-related integrins may be able to stimulate the beta-catenin pathway [22, 28]. In order to confirm a specific role of α5β1 integrin in the fibronectin-dependent activation of beta-catenin, we next compared the activation process in U87MG cells with α5 high or low expression. Fibronectin-dependent beta-catenin activation was strongly enhanced in α5-high cells. In α5-low cells the low basal activity of beta-catenin was enhanced by fibronectin until reaching the basal level in α5-high cells (Figure 2B). Similar results were obtained in U373MG cells (Figure 2C). Data thus confirmed that on a fibronectin matrix, beta-catenin activation occurs upon fibronectin-linked α5 integrin activation but do not exclude participation of other fibronectin receptors (such as αvβ3 integrin which is also expressed on U87MG and U373MG cells).

### Integrin α5β1 activation increases β-catenin transactivation in glioma cells

**Figure 3:**
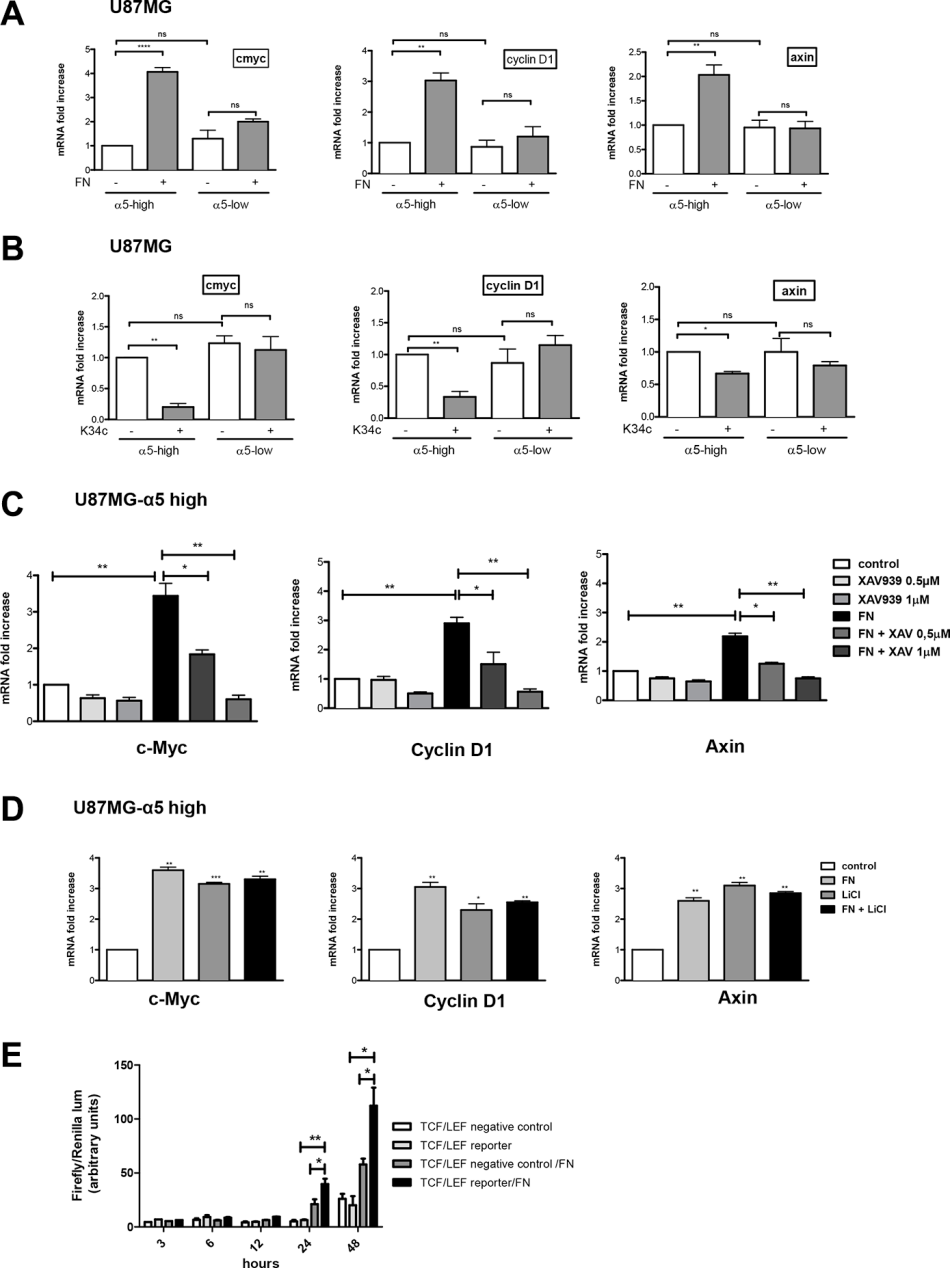
Integrin α5β1 activation increases β-catenin transactivation in U87MG cells Transcriptional activity of β-catenin was recorded by mRNA variation analysis (RT-qPCR) of downstream known targets of beta-catenin (cmyc, cyclin D1 and axin). (**A**) U87MG α5-high and α5-low cells were incubated during 6 hours on uncoated or fibronectin–coated wells. (**B**) U87MG α5-high and α5-low cells were incubated during 6 hours on uncoated wells with or without the integrin antagonist K34c (20 μM). (**C**) U87MG α5-high cells were incubated during 6 hours on uncoated wells as the control condition and compared with cells on fibronectin-coated wells (10 μg/ml) in the absence or presence of XAV939 (0.5 and 1 μM), a tankyrase inhibitor which provokes the degradation of β-catenin. (**D**) U87MG α5-high cells were incubated during 6 hours on uncoated wells as the control condition, on fibronectin-coated wells (10 μg/ml), in the presence of lithium chloride (LiCl, 30 μM; a known activator of β-catenin transactivation) or both. (**E**) TCF/LEF reporter activity of U87MG-α5 high cells plated on fibronectin. Cells were transfected with a negative control or an inducible transcription factor responsive firefly luciferase reporter and a constitutively expressing Renilla construct and plated on uncoated or fibronectin-coated wells for the times indicated. Histograms represent relative luminescence units (firefly/renilla arbitrary units). Data represent the mean ± S.E.M. of 3 independent experiments with *p < 0,05; **p < 0,01; ***p < 0,005; ns, non-significant.

In the former assays, beta-catenin activation was determined by mean of protein level with a specific anti-active beta-catenin antibody [27]. Activation process of beta-catenin was next investigated on the transcriptional activity level. Downstream known targets of beta-catenin transactivation, c-myc, cyclin D1 and axin, were analyzed by real time PCR after cell plating on fibronectin. Interestingly, although basal mRNA level of the 3 genes was not affected by the expression level of α5 integrin, fibronectin clearly enhanced their transcription in a α5 integrin-dependent manner for both U87MG (Figure 3A) and U373MG cells (Supplementary Figure S1A). Conversely, inhibition of α5β1 integrin activity by K34c only affected negatively the mRNA level of the 3 genes in U87MG- and U373MG-α5 high cells (Figure 3B and Supplementary Figure S1B). Data thus suggested that transcriptional activation of beta-catenin was only obtainable in an α5 integrin–dependent way. To further confirm the implication of the beta-catenin pathway in these effects, U87MG-α5 high cells were treated with a tankyrase inhibitor, XAV939, which is known to promote beta-catenin degradation [29] See Figure 5A). The fibronectin-induced increase of gene transcription was highly and dose-dependently downregulated by XAV939 (Figure 3C). In addition, U87MG-α5 high cell treatment with LiCl, a known inducer of beta-catenin transactivation, increased the gene transcription up to the level obtained with fibronectin (Figure 3D) whereas treatment with both compounds did not enhance this effect. Finally, we confirmed that α5 integrin activation by fibronectin led to the activation of the beta-catenin/TCF/LEF signaling pathway as shown in the Figure 3E. Taken together, data indicated that only fibronectin-dependent activation of α5β1 integrin led to transcriptional activity of beta-catenin in glioma cell lines.

### Integrin α5–dependent beta-catenin activation is not implicated in glioma cell survival and resistance to p53 activators

**Figure 4:**
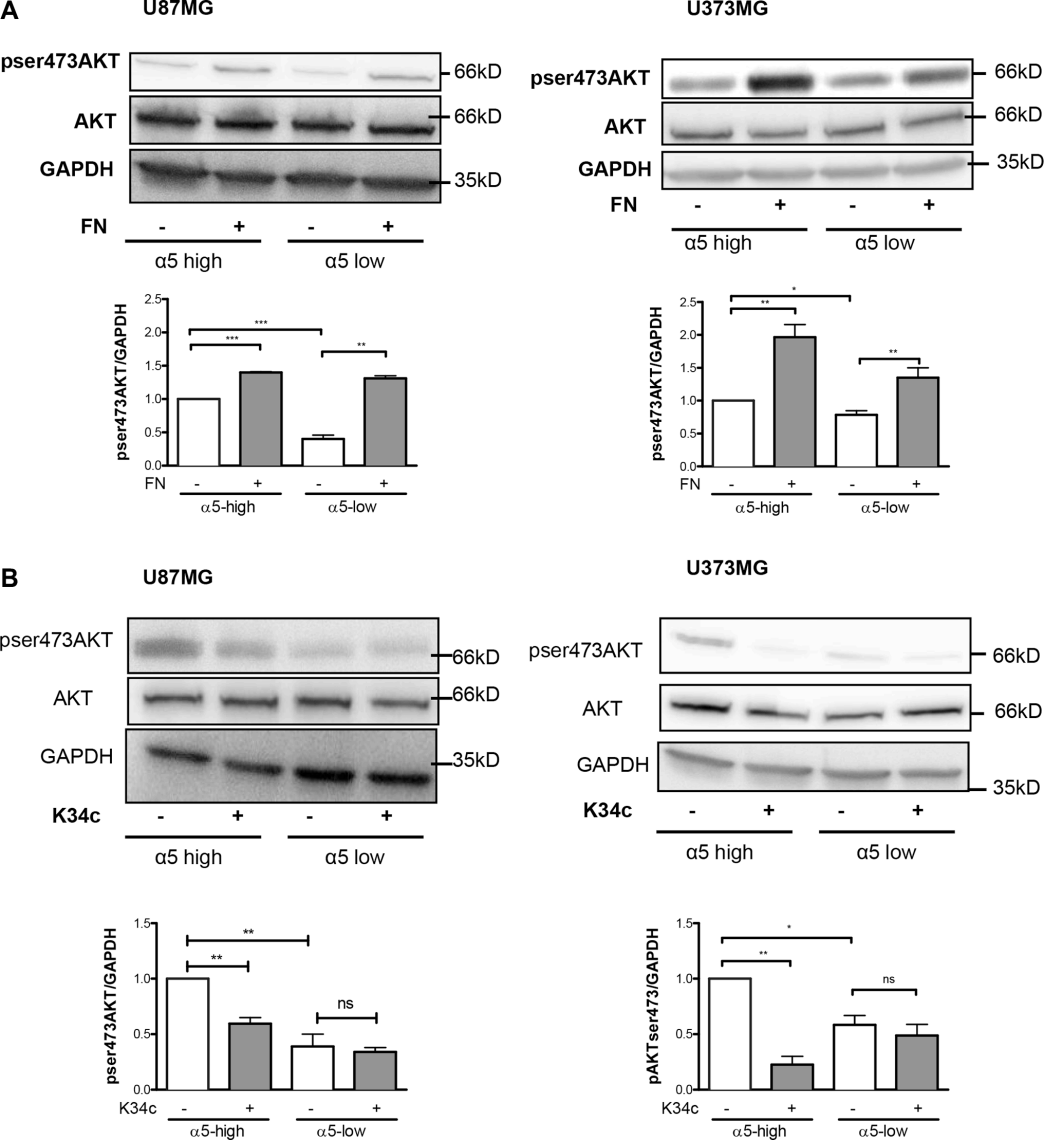
Expression of α5β1 integrin modulates the activity of AKT in U87MG and U373MG cell lines Western blot analysis of AKT activation in α5 high and α5 low glioma cells plated for 90 min on fibronectin-coated wells (10 μg/ml) (**A**) or treated with K34c (20 μM) (**B**). GAPDH was used as loading control. Histograms show the fold increase in active AKT as compared to those obtained on uncoated wells of at least three independent experiments. Data represent the mean ± S.E.M. of 3 independent experiments with *p < 0,05; **p < 0,01; ***p < 0,005; ns, non-significant.

**Figure 5:**
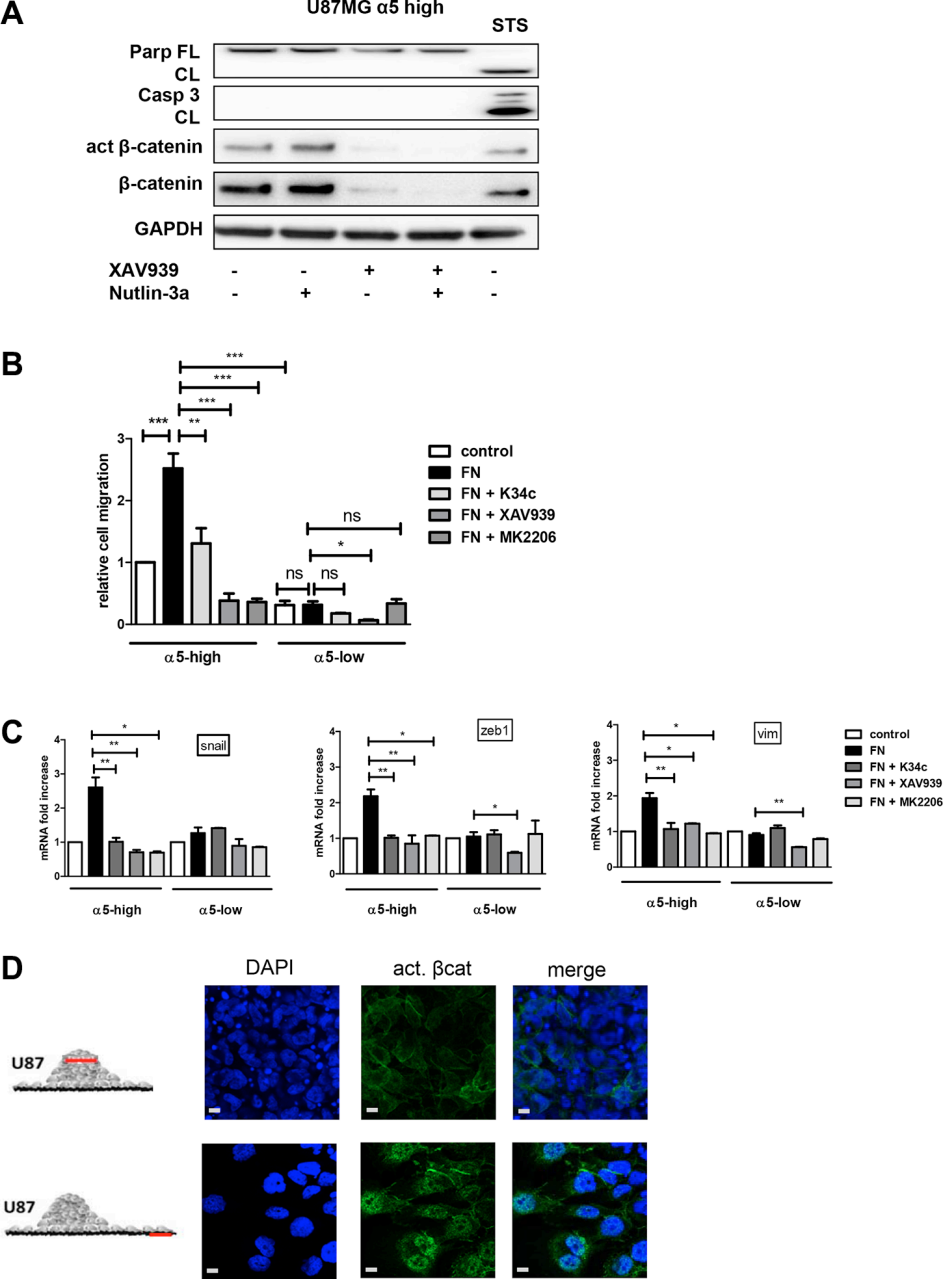
α5β1 integrin-dependent β-catenin activation triggers cell migration and not resistance to therapy scale bar represent 10 μm (**A**) U87MG α5-high cells were treated during 12 hours with Nutlin-3a (a p53 activator- 5 μM), with XAV939 (a tankyrase inhibitor inducing β-catenin degradation −1 μM)) or with both. Protein extracts were analyzed for apoptosis markers (cleaved PARP and cleaved caspase3) by immunoblots. Staurosporine (STS - 1μM) was used in parallel as a control of functional apoptotic machinery in the cells. One western blot representative of at least 3 experiments is shown. (**B**) Migration of U87MG α5-high cells was examined by the Boyden Chamber assay. Cells were plated on the upper side of the chambers either uncoated (control) or coated with fibronectin (FN −10 μg/ml) and treated with the integrin antagonist K34c (20 μM), the tankyrase inhibitor XAV939 (1 μM) or the AKT inhibitor MK2206 (20 μM); migration was allowed during 24 hours. Relative cell number at the bottom side of the chamber, for each condition as compared to the control condition, is reported on the histograms. (**C**) Transcriptional activity of β-catenin was recorded in U87MG α5-high and α5-low cells by mRNA variation analysis (RT-qPCR) of downstream known targets of beta-catenin implicated in epithelial to mesenchymal transition (EMT), snail, zeb1, vimentin (vim). Cells were plated during 6 hours on uncoated wells as the control condition and compared with cells on fibronectin-coated wells (10 μg/ml) in the absence or presence of K34c (20μM), XAV939 (1 μM) or MK2206 (20 μM). (**D**) Two-day-old U87MG-α5 high spheroids were plated on FN-coated (10 μg/mL) glass coverslips. 18 hours later, cells were fixed and active beta-catenin was immunodetected. Confocal images were taken at 2 different zones as depicted in the diagrams to examine (top images) cells in the tumor sphere at a focal plane 5 μm above the coverslips and (bottom images) cells that migrated away from the sphere. Data represent the mean ± S.E.M. of 3 independent experiments with *p < 0,05; **p < 0,01; ***p < 0,005.

We already demonstrated the implication of α5 integrin in glioma chemoresistance and tumor cell migration. Our recent data implicated the integrin-dependent PI3K/AKT pathway activation in resistance to p53 activators. Inactivation of AKT via integrin antagonist K34c was required to sensitize U87MG-α5 high cells to p53 activator Nutlin-3a [21]. As inactivation of PI3K/AKT signaling is known to modulate beta-catenin-mediated gene transcription [30–32], we wondered if integrin-driven AKT pathway may be involved in beta-catenin activation. Data shown in Figure 4A and 4B indicate that AKT phosphorylation on Ser473 (indicative of its activation) is induced on fibronectin and inhibited by K34c in U87MG- and U373MG-α5 high cells looking like the results obtained for beta-catenin activation (Figure 1A). Additionally, basal activity of AKT was lower in both U87MG- and U373MG- α5 low cells and no further inhibitory effect was obtained with K34c in these cells (Figure 4B). Data suggest a relationship between AKT and active beta-catenin supported by α5 integrin expression levels. If such, beta-catenin inhibition may act in synergy with Nutlin-3a in the purpose of killing the tumoral cells. To evaluate the potential of α5/AKT-dependent beta-catenin implication in resistance to p53 activators, we treated U87MG-α5 high cells with XAV939 and Nutlin-3a and recorded markers of apoptosis. As shown in the Figure 5A, no apoptosis could be obtained with this co-treatment in contrast to those obtained with the combination of the integrin antagonist K34c and Nutlin-3a [21]. According to these data, it seems that beta-catenin activation by α5β1 integrin/AKT pathway does not trigger cell survival and resistance to therapy in our experimental conditions.

### Integrin α5–dependent beta-catenin activation is implicated in glioma cell migration

In addition to its role in proliferation or survival, beta-catenin also impacts on cell migration [33]. We already have shown that α5 integrin expression in glioma cells triggers single cell migration which is blocked by specific antagonists [19]. To uncover a potential relationship between α5β1 integrin-driven migration and beta-catenin activation we evaluated cell migration of U87MG-α5 high and low cells by Boyden Chamber assays. Fibronectin coating clearly enhanced the migration of U87MG-α5 high cells without affecting those of U87MG-α5 low cells (Figure 5B). K34c antagonist significantly decreased the fibronectin/α5-dependent cell migration (Figure 5B), without affecting the U87MG-α5 low cell migration. Cell treatment with XAV939 also clearly inhibited the α5-driven fibronectin-dependent cell migration (Figure 5B) supporting an important role of beta-catenin activation in cell migration. Importantly, MK2206, an AKT inhibitor, behave similarly as K34c and XAV939 and also inhibited U87MG-α5 high cell fibronectin-dependent migration (Figure 5B). In line with this, the beta-catenin-identified targets implicated in EMT-induced cell migration snail, vimentin and zeb1 mRNAs were increased in U87MG-α5 high cells plated on fibronectin and decreased by K34c, XAV939 or MK2206 treatments (Figure 5C). We confirmed that vimentin was similarly affected at the protein level (Supplementary Figure S2). In U87MG-α5 low cells, neither migration nor snail, vimentin or zeb1 mRNA levels were affected by fibronectin, K34c or MK2206 (Figure 5B and 5C). Only XAV939 inhibited, in a non α5 integrin–dependent way, migration and gene levels in U87MG-α5 low cells. Similar data have been obtained in U373MG cell line (Supplementary Figure S3). To confirm the relationship between α5β1 integrin-driven migration and beta-catenin activation, we used the spheroid model of U87MG-α5 high cells we described recently [34]. In this model, α5β1 integrin is involved in cell-cell cohesion inside the spheroid but drives cell migration out of the spheroid on fibronectin-coated surfaces. As shown in the Figure 5D, active beta-catenin was clearly localized only at the cell membrane in the spheroid but relocalized to the nucleus in migrating cells. Data thus suggest that translocation of beta-catenin to the nucleus leading to its transactivation may drive α5β1 integrin-dependent migration.

Taken together, data show that α5 integrin activation triggers AKT and beta-catenin stimulation, inducing an EMT-like program and cell migration.

## DISCUSSION

We present here data suggesting that α5β1 integrin is to be considered as a new important driver of beta-catenin activation leading mostly to tumor cell migration through EMT-like processes. Aberrant activation of the Wnt/beta-catenin pathway has been largely documented in brain tumors where it contributes to the maintenance of stem-like cells, resistance to therapies and to an invasive phenotype [6]. Unlike in other tumor types, no activating mutations of beta-catenin gene (CTNNB1) were detected in glioma excluding these events as drivers of cytoplasmic stabilization of beta-catenin [5, 35]. Dysregulation of the Wnt canonical pathway leading to beta-catenin activation has been detected in glioma patients with overexpression of WNT3a [5] and epigenetic-driven downregulation of inhibitors like SFRP1, Dkk1 or Wif1 [36, 37]. Wnt/beta-catenin canonical pathway activation has been associated positively with increasing tumor grades and poor glioma patient survival [4, 38]. Wnt-independent mechanisms of beta-catenin activation also exist in glioma including the EGF/EGFR pathway [12]. Knowledges about beta-catenin regulation in glioma are recently increasing with R132H IDH1 mutation [39], FoxM1 [40], TRIM33 [41], HOXA13 [42] as examples of positive or negative regulators. Based on our data, α5β1 integrin has to be added to this growing list of beta-catenin regulators in glioma cells.

Expression level (mRNA and protein) of beta-catenin is considered as a negative prognostic marker in glioma with an enhanced expression in glioblastoma [8, 38]. Interestingly we and others have shown that α5 integrin mRNA level is associated with high glioma grade and a bad patient prognosis [16]. In our current experiments, we have shown that variations in the α5β1 integrin expression did not alter the basal level of beta-catenin protein but modulated an active conformation of beta-catenin (Figure 1). The antibodies we used in western blot experiments were described to specifically recognize a non-phosphorylated Ser37/Thr41 N-terminal beta-catenin epitope generated upon Wnt signaling or GSK3 inhibition [27]. Our results thus suggest that α5 integrin may be involved in the stabilization of a fraction of beta-catenin in a non-degradable form. However, our data have also shown that α5 integrin overexpression alone did not allow the transcriptional activation of beta-catenin. Only integrin activation through fibronectin clearly provoked a beta-catenin transactivation. The specific implication of α5β1 integrin in the fibronectin-dependent beta-catenin activation was further confirmed by using α5-depleted cells and a specific α5β1 integrin antagonist, K34c. According to our data, we propose for the first time that α5β1 integrin plays a dual role in glioma beta-catenin pathway, one presumably linked to its expression level resulting to a stabilization of beta-catenin and the other only achieved after integrin activation leading to beta-catenin transactivation. Further explorations are nevertheless needed to clearly define the fine tuning of this machinery and the potential contributions of Wnt-dependent and independent pathways. We can however already hypothesize that concomitant high expression of beta-catenin and α5 integrin in high grade glioma will strengthen their aggressiveness.

**Figure 6:**
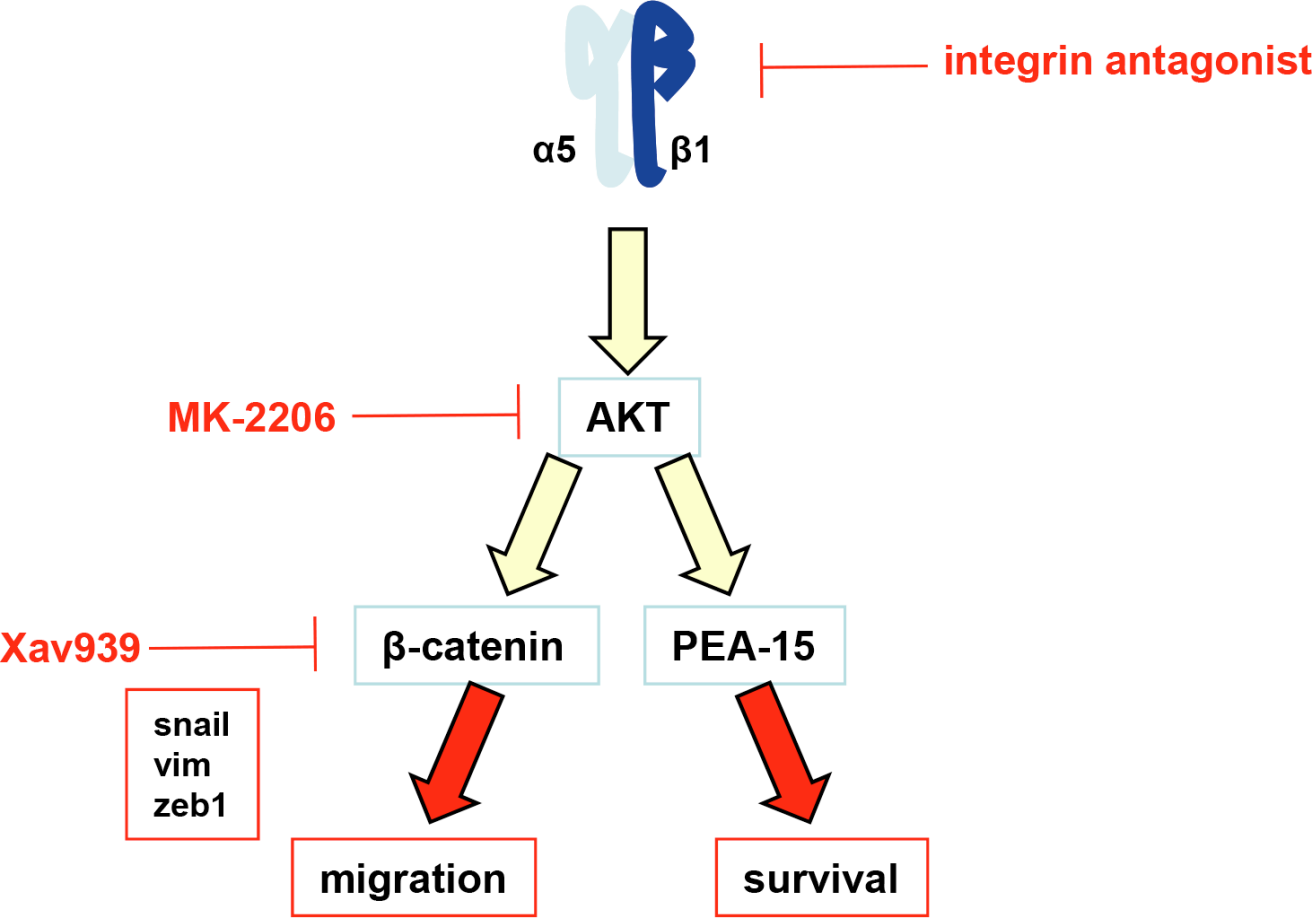
Schematic representation of the α5β1 integrin/AKT pathway in glioma cells The α5β1 integrin overexpression or activation induces the activation of AKT. We have shown recently that AKT modulates the PEA-15 anti-apoptotic protein to impair the p53-induced pro-apoptotic pathway [21]. According to the present work, we now have shown that activation of α5β1 integrin by fibronectin also led to AKT activation followed by transactivation of beta-catenin, induction of an EMT-like program and an increase in cell migration.

Among the hallmarks of glioblastoma, resistance to apoptosis and invasion into the normal brain are both implicated in their propensity to resist standard therapies. As reviewed in Schaffner et al. [43] and Blandin et al. [18], α5β1 integrin has implications in both processes for various solid tumors. We have shown elsewhere that α5 integrin-dependent pro-survival signaling depended on the AKT pathway [21]. Our present results show that this integrin-dependent AKT pathway is also involved in glioma cell migration (Figure 5). Phosphorylation of Ser552 on beta-catenin by AKT activation promotes its transcriptional activity [30] and accelerates migration and invasion [33]. We demonstrated here that α5β1 integrin stimulation triggers both AKT and beta-catenin activation leading to cell migration, which are all inhibited by an integrin antagonist. Our data thus define a new α5β1 integrin/AKT/beta-catenin pathway implicated in glioma cell migration. A point of intersection between integrin pro-survival or pro-migratory pathways is therefore achieved through AKT (Figure 6) which is largely involved in glioma malignancy [44]. Interestingly, in our experimental conditions, molecular determinants for these pathways diverge as reducing beta-catenin expression by XAV939 did not synergize with the p53 activator, Nutlin-3a, to induce apoptosis (Figure 5A). Data are in line with those of Kahlert et al. [9], showing that activation of canonical Wnt/beta-catenin in glioma cells (including U87MG cells) enhances *in vitro* motility but not proliferation. Interestingly, these authors also showed that nuclear (active) beta-catenin was predominantly found in the infiltrative zone of glioblastoma supporting its role in cell migration *in vivo*.

In solid tumors, acquisition of a migratory phenotype is often the result of an epithelial to mesenchymal transition (EMT). EMT-like processes in glioma (of non-epithelial origin) have recently been described [45] and a “mesenchymal transition metagene” proposed, which included fibronectin, the ligand of α5β1 integrin [46]. A mesenchymal subtype of glioblastoma was associated with aggressiveness and disease progression as reported in integrated genomic analysis [47, 48]. Our study clearly shows that an EMT-like transition is obtained by fibronectin-dependent activation of α5β1 integrin in glioma cells as known EMT activators (snail, zeb1) or markers (vim) are increased under the control of beta-catenin and decreased by the integrin antagonist K34c. Interestingly, α5 integrin was part of a six mesenchymal gene signature in carcinoma EMT [49]. Similarly, recent works highlighted the regulation of beta-catenin signaling by α2/α3β1 integrin clustering in epithelial ovarian carcinoma resulting in increased invasion and metastasis [22, 50]. Promotion of EMT was also assigned to α9β1 integrin in colon and lung carcinoma [25]. Our data thus add new evidences that β1 subunit-containing integrins play a crucial role in EMT induction and migration of tumoral cells out of the primary tumor site even in glioma.

## MATERIALS AND METHODS

### Cell lines and compounds

U87MG and U373MG cells were obtained from ATCC and ECACC respectively several years ago. Cells were manipulated genetically to overexpress (U87MG- and U373MG-α5 high cells) or repress (U87MG- and U373MG-α5 low cells) the α5 integrin subunit as described earlier [16, 21]. Briefly, cells were stably transfected to overexpress (by transfecting a pcDNA3.1 plasmid containing the human a5 integrin gene; provided by Dr. Ruoshlati, University of California, Santa Barbara, CA) or to repress [by transfecting a pSM2 plasmid coding for a short hairpin RNA (shRNA) targeting the a5 mRNA; Open Biosystems] the a5 integrin subunit by using jetPRIME (Polyplus transfection) according to the manufacturer's instructions. LN443, LN18 and LN319 glioma cells were kindly provided by Pr M. Hegi (Lausanne, Switzerland). Early passage (< 10) patient-derived glioblastoma cell line T20 was obtained from Dr J. Nuesch (DKFZ, Heidelberg, Germany). Cells were not authenticated in the laboratory but regularly checked for expression level of α5 integrin and p53 status (by NGS) to compare with the original cells. Cells were routinely cultured in DMEM or EMEM supplemented with 10% heat-inactivated FBS, and 200IU/mL penicilin/streptomycin. Small non peptidic specific α5β1 integrin antagonist K34C has been described elsewhere [19]. LiCl and Xav939 were from Sigma. MK-2206 was from CliniSciences. Drugs were prepared as stock solutions in DMSO at 10 mM and kept at −20°C until use. DAPI was purchased from Santa Cruz Biotechnology. Human fibronectin stabilized solution was kindly provided by Pr Carreiras, (EA1391, Cergy Pontoise). Vitronectin was a gift from Pr J. Luis (Marseille, France). Collagen, laminin and poly-L-Lysine were from Sigma. Cell culture medium and reagents were from Lonza.

### Cell handling

To measure basal levels of signaling pathways (i.e. in conditions where integrins are not exogenously activated by serum components), trypsinized cells were washed 3 times in serum free medium by centrifugation and then plated (200,000 cells/well, 6-well plastic plates) in the presence of drugs or controls (DMSO 0.2%). When required, cells were plated on substrate-coated dishes (fibronectin, vitronectin, collagen, laminin or poly-L-lysine - 10 μg/mL) and incubated for 90 minutes (western blot analysis) or 6 hours (RTq-PCR analysis) in a humidified incubator containing 5% CO_2_ at 37°C.

### Migration assay

Cell migration was analyzed by a Boyden Chamber assay using an 8 μm pore size Transwell permeable support (24-well Transwell plates, Millipore). The bottom part of the Transwell membrane was either uncoated or coated with fibronectin. Fibronectin coating (10 μg/mL in phosphate buffer saline) was achieved at 37°C in a CO_2_ incubator for 3 hours. The coated and uncoated chambers were then placed in the 24-well plates filled with respective culture medium supplemented with the drugs as required. Cells (1 × 104 cells/chamber) in culture medium containing or not the required drugs were seeded into the upper part of the chamber and the plates were transferred in a humidied incubator containing 5% CO_2_ at 37°C. After 24 h of incubation, cells that reached the bottom layer were fixed with paraformaldehyde (4%) and stained with DAPI. Fluorescent images were acquired under a Zeiss Axio microscope under 10× magnification (6 views field) and cell number quantified with a home-made macro using the ImageJ software.

### Western blotting

Proteins were separated on precast gradient 4–20% SDS PAGE gels (Biorad) and transferred to PVDF membrane (Amersham Bioscience) under established protocol settings. After blocking, membranes were probed with primary antibodies (1/1000) against α5 integrin (HI04-Santa-Cruz; sc-10729), β1 integrin (TS2/16 - Santa-Cruz; sc53711), fibronectin (BD transduction laboratories; #610077), (non-phospho Ser33/37/Thr41) active β-catenin (8E7, Millipore; #05665), β-catenin (Millipore. #06-734), GAPDH (Millipore; #AB2302), AKT (Cell Signaling Technology; #9272), pSer473-AKT (D9E; Cell Signaling Technology; #4060), vimentin (Santa-Cruz; sc-6260), cleaved caspase 3 (Cell Signaling Technology; #9604) and PARP (BD transduction laboratories; #556494) antibodies. Immunological complexes were revealed with HRP-conjugated secondary antibodies (Promega) at 1/10 000 dilution using chemoluminescence (ECL, Biorad) and visualized with Las4000 image analyser (GE Healthcare). Quantification of non-saturated images was performed with ImageJ software. GAPDH was used as the loading control for all samples.

### Real-time PCR analysis

RNA extracted with RNeasy minikit (Qiagen), according to the manufacturer protocol, was transcribed into cDNA using the high capacity cDNA kit (Applied Biosystem). Real time quantitative PCR was performed using the Fast SYBR Green Master Mix and the StepOne Plus Real time PCR system (Applied Biosystem) (for primer sequences, see Supplementary Table S1). Target cDNA expression was quantified using the comparative ΔΔCt method with GAPDH or cyclophilin as internal controls with similar results.

### TCF/LEF luciferase reporter assay

Cells plated were transiently transfected using Lipofectamine 2000 (Invitrogen) with a mixture of an inducible transcription factor responsive firefly luciferase reporter vector or a non inducible vector and a constitutively expressing Renilla construct to normalize for transfection efficiency (Cignal reporter assay kit, Qiagen). Three independent transfections were performed. Following transfection, cells in serum free medium were plated on uncoated or fibronectin-coated wells during 3, 6, 12, 24 or 48 hours. Lysates were harvested, and luciferase activities were recorded with a Dual-Luciferase^®^ Reporter Assay System (Promega) according to the manufacturer's instructions. Luciferase activity was monitored with a Multidetection Microplate Reader (TriStar^2^ LB 942 − Berthold Technologies).

### Spheroids formation and immunolabeling

Spheroids were formed and analysed as described in Blandin et al. [34]. Briefly, single cell suspension was generated from trypsinized monolayers and diluted at the desired cell density. Cell suspension was mixed in EMEM/10%FBS containing 20% of methylcellulose. Cell spheroids were formed by the hanging drop method (25 μL, 2000 cells per sphere). Two-day-old spheroids were seeded on fibronectin-coated glass coverslips for 18 hours. Cells were then fixed in 3.7% paraformaldehyde, and permeabilized with 0.1% Triton-X100. After a 1 hour blocking step using TBS-BSA 3% solution, cells were incubated with anti-active beta-catenin primary antibody O/N at 4°C (2 μg/mL in TBS-BSA 3%) and secondary antibody (Alexafluor 488 goat anti-mouse IgG - #A11001, Life Technologies) and DAPI for 1 hour. Spheroids were mounted on microscope slides using Permafluor Aqueous Mounting Medium (Fisher). Images were acquired using a confocal microscope (LEICA TCS SPE II, 60× magnification oil-immersion).

### Statistical analysis

Data are represented as the mean ± SEM, and n is the number of independent experiments. Statistical analyses were conducted using the Student *t* test with the GraphPad Prism program. *P* < 0.05 was considered significant.

## CONCLUSIONS

Taken together, our results show for the first time that the α5β1 integrin modulates the beta-catenin pathway in glioma to sustain an EMT-like based migratory program. In addition with our previous results, the interplay we describe here highlights the potential pivotal role of the α5β1 integrin in hallmarks of brain tumors, i.e. resistance to apoptosis and invasion in the normal brain. Antagonists of this specific integrin may thus be proposed as new options for helping to block both processes in subpopulations of high grade glioma.

## SUPPLEMENTARY MATERIALS FIGURES AND TABLE


