# Supplementary material for: Expression/activation of α5β1 integrin is linked to the β-catenin signaling pathway to drive migration in glioma cells
Source: Oncotarget. 2016 Aug 23;7(38):62194–207. doi: 10.18632/oncotarget.11552 (PMC5308720; doi:10.18632/oncotarget.11552)
Supplement: Supplementary file 1 [file oncotarget-07-62194-s001.pdf]

Expression/activation of α5β1 integrin is linked to the β-catenin signaling pathway to drive migration in glioma cells

Supplementary Materials

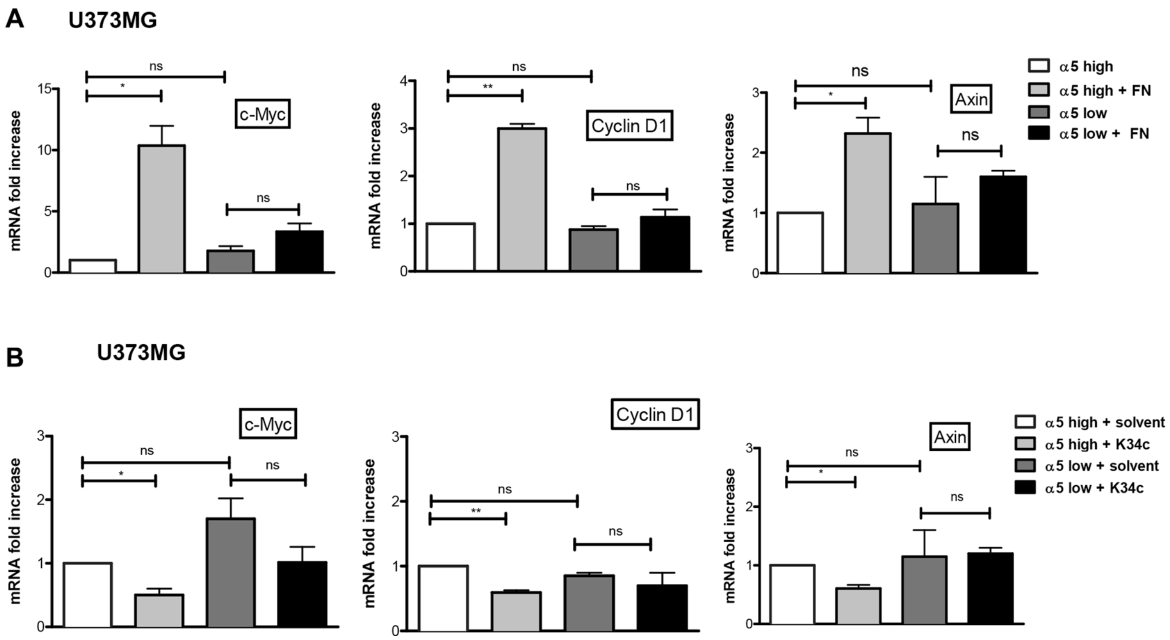

Supplementary Figure S1: Integrin α5β1 activation increases β-catenin transactivation in U373MG cells. Transcriptional activity of β-catenin was recorded by mRNA variation analysis (RT-qPCR) of downstream known targets of beta-catenin (c-myc, cyclin D1 and axin). (**A**) U373MG α5-high and α5-low cells were incubated during 6 hours on uncoated or fibronectin–coated wells. (**B**) U373MG α5-high and α5-low cells were incubated during 6 hours on uncoated wells with or without the integrin antagonist K34c (20 µM). Data represent the mean ± S.E.M. of 3 independent experiments with *$p < 0.05$; **$p < 0,01$; ns, non-significant.

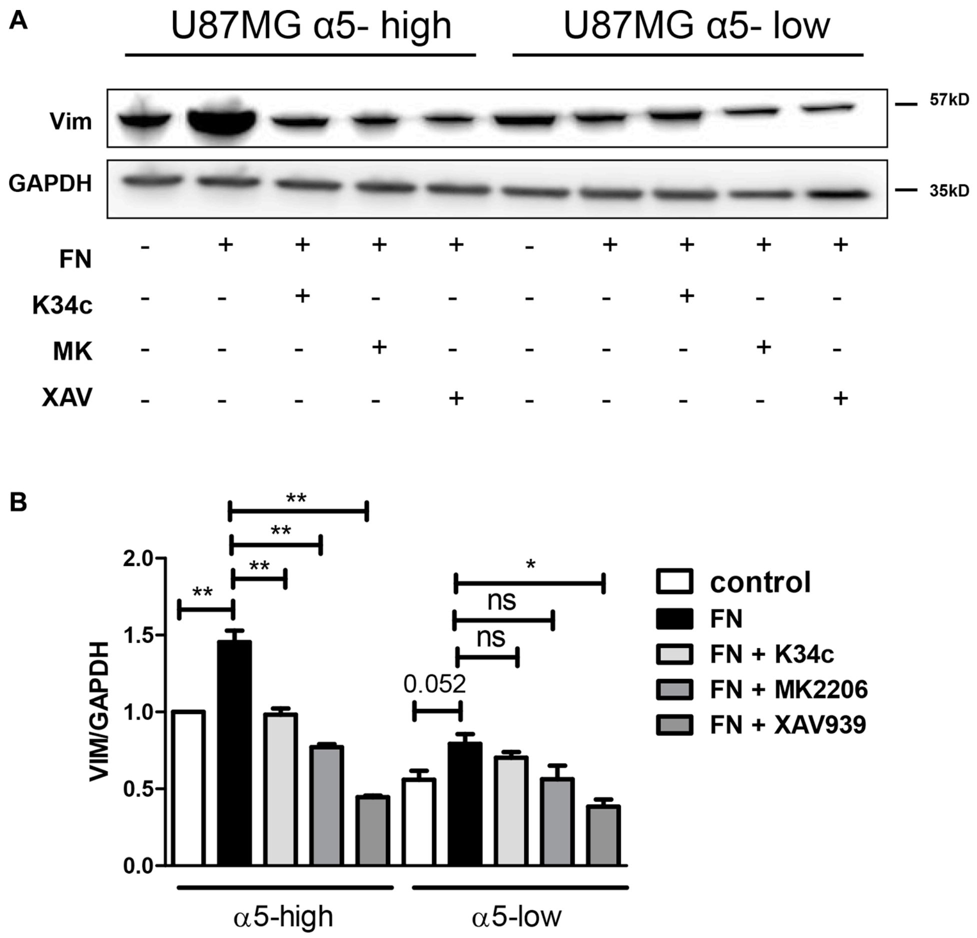

Supplementary Figure S2: Vimentin protein level is modulated similarly to vimentin mRNA level in U87MG-α5 cells. Cells were plated during 6 hours on uncoated wells as the control condition and compared with cells on fibronectin-coated wells (10 µg/ml) in the absence or presence of K34c (20 µM), XAV939 (1 µM) or MK2206 (20 µM). Vimentin expression level variations were recorded by western blot analysis with a specific antibody (Santa Cruz; sc-6260). (**A**) One western blot representative of at least 3 experiments is shown. (**B**) Data represent the mean ± S.E.M. of 3 independent experiments with *$p < 0.05$; ** $p < 0.01$; ns, non-significant.

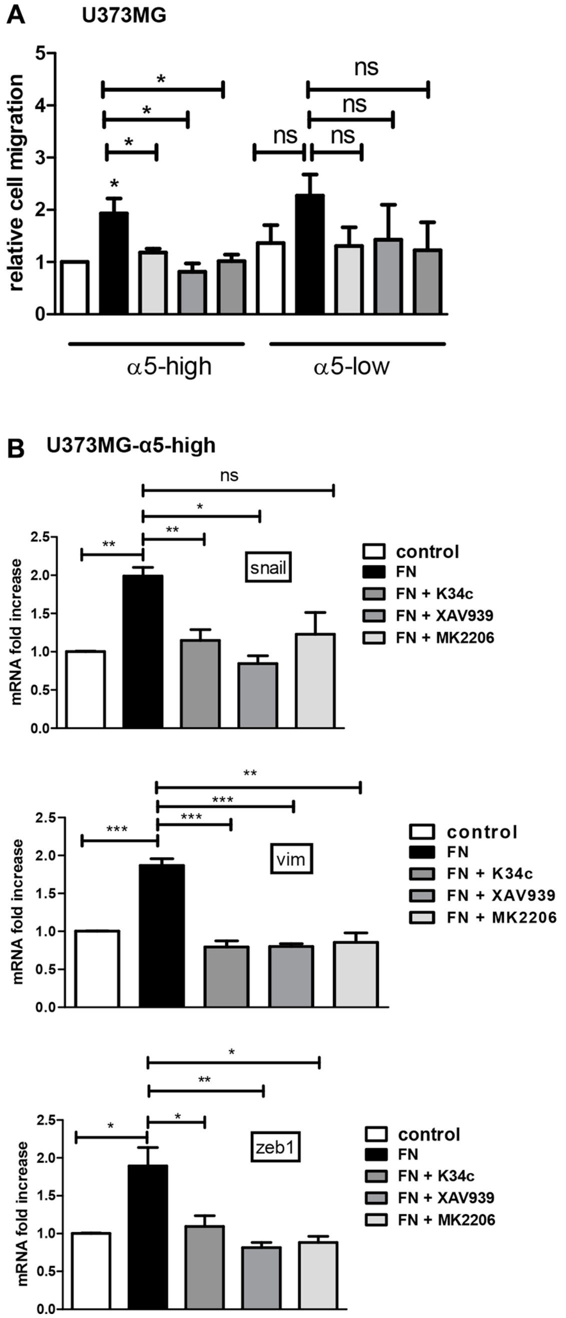

Supplementary Figure S3: α5β1 integrin-dependent β-catenin activation triggers cell migration in U373MG cells through EMT-like process. (**A**) Migration of U373MG α5-high cells was examined by the Boyden Chamber assay. Cells were plated on the upper side of the chambers either uncoated (control) or coated with fibronectin (FN -10 µg/ml) and treated with the integrin antagonist K34c (20 µM), the tankyrase inhibitor XAV939 (1 µM) or the AKT inhibitor MK2206 (20 µM); migration was allowed during 24 hours. Relative cell number at the bottom side of the chamber, for each condition as compared to the control condition, is reported on the histograms. (**B**) Transcriptional activity of β-catenin was recorded in U373MG α5-high cells by mRNA variation analysis (RT-qPCR) of downstream known targets of beta-catenin implicated in epithelial to mesenchymal transition (EMT), snail, zeb1, vimentin (vim). Cells were plated during 6 hours on uncoated wells as the control condition and compared with cells on fibronectin-coated wells (10 µg/ml) in the absence or presence of K34c (20 µM), XAV939 (1 µM) or MK2206 (20 µM). Data represent the mean ± S.E.M. of 3 independent experiments with *$p < 0.05$; **$p < 0.01$; ***$p < 0.005$; ns, non-significant.

Supplementary Table S1: list of the primers used in the study

Gene	Forward 5′- 3′	Reverse 3′ - 5′
GAPDH	GTCACCAGGGCTGCTTTTAACTCT	GCAATCATATTGGAACATGTAAACCA
C-myc	CTTGTTGCGGAAACGACGAG	ACTCAGCCAAGGTTGTGAGG
CyclinD1	GCTGTGCATCTACACCGACA	TTGAGCTTGTTCACCAGGAG
Axin	GGAGAGCGTGCAGGTCAAT	CACAGCCCATGTCCACACA
Zeb1	GAAAGTGATCCAGCCAAATGGAA	TTTGGGCGGTGTAGAATCAGAG
Snail	GACCACTATGCCGCGCTCTT	TCGCTGTAGTTAGGCTTCCGATT
Vimentin	TGAGTACCGGAGACAGGTGCAG	TAGCAGCTTCAACGGCAAAGTTC
